# Imaging grafted cells with [^18^F]FHBG using an optimized HSV1-TK mammalian expression vector in a brain injury rodent model

**DOI:** 10.1371/journal.pone.0184630

**Published:** 2017-09-19

**Authors:** Anne-Sophie Salabert, Laurence Vaysse, Marie Beaurain, Mathieu Alonso, Germain Arribarat, Jean-Albert Lotterie, Isabelle Loubinoux, Mathieu Tafani, Pierre Payoux

**Affiliations:** 1 ToNIC, Toulouse NeuroImaging Centre UMR1214, Université de Toulouse, Inserm, UPS, France; 2 University hospital, Radiopharmacy Unit, Toulouse, France; 3 University hospital, Nuclear medecine Unit, Toulouse, France; Wayne State University, UNITED STATES

## Abstract

**Introduction:**

Cell transplantation is an innovative therapeutic approach after brain injury to compensate for tissue damage. To have real-time longitudinal monitoring of intracerebrally grafted cells, we explored the feasibility of a molecular imaging approach using thymidine kinase HSV1-TK gene encoding and [^18^F]FHBG as a reporter probe to image enzyme expression.

**Methods:**

A stable neuronal cell line expressing HSV1-TK was developed with an optimised mammalian expression vector to ensure long-term transgene expression. After [^18^F]FHBG incubation under defined parameters, calibration ranges from 1 X 10^4^ to 3 X 10^6^ Neuro2A-TK cells were analysed by gamma counter or by PET-camera. In parallel, grafting with different quantities of [^18^F]FHBG prelabelled Neuro2A-TK cells was carried out in a rat brain injury model induced by stereotaxic injection of malonate toxin. Image acquisition of the rats was then performed with PET/CT camera to study the [^18^F]FHBG signal of transplanted cells *in vivo*.

**Results:**

Under the optimised incubation conditions, [^18^F]FHBG cell uptake rate was around 2.52%. *In-vitro* calibration range analysis shows a clear linear correlation between the number of cells and the signal intensity. The PET signal emitted into rat brain correlated well with the number of cells injected and the number of surviving grafted cells was recorded via the *in-vitro* calibration range. PET/CT acquisitions also allowed validation of the stereotaxic injection procedure. Technique sensitivity was evaluated under 5 X 10^4^ grafted cells *in vivo*. No [^18^F]FHBG or [^18^F]metabolite release was observed showing a stable cell uptake even 2 h post-graft.

**Conclusion:**

The development of this kind of approach will allow grafting to be controlled and ensure longitudinal follow-up of cell viability and biodistribution after intracerebral injection.

## Introduction

Cell transplantation is an innovative therapeutic approach, particularly after brain injury, to compensate for tissue damage and neuronal loss. However, before translation to the clinic, we need to have a better understanding of underlying graft mechanisms and ensure the safety of this kind of approach [1].

To ensure real-time longitudinal monitoring of cell grafts, a molecular imaging approach was recently developed using thymidine kinase (HSV1-TK) gene encoding and [^18^F]FHBG as a reporter probe to image enzyme expression [2–4]. Selective monophosphorylation of nucleoside analogues by virus-encoded thymidine kinase has long since been used in suicide gene therapy for cancer [5]. Similarly, HSV1-TK specifically phosphorylates the radiotracer [^18^F]FHBG leading to its intracellular accumulation only in cells previously genetically modified to express HSV1-TK. Other fluorine probes for imaging HSV1-TK activity have been described such as [^18^F]- FIAU. However [^18^F]-FHBG seems to afford optimum cell uptake [6]. It also has the advantage of being a well-known radiotracer with a widely accessible synthesis precursor.

[^18^F]FHBG is a PET imaging radiotracer that is easily transferrable to human applications [7,8]. PET scans allow radiolabelled markers and their interactions with biochemical processes to be imaged in living subjects. Given this incredible nanomolar (<10^-9^M) sensitivity, PET is capable of measuring biological processes at very low concentrations [9]. Moreover, PET molecular imaging could provide effective brain imaging as opposed to optical imaging as bioluminescence is still limited by a lower spatial resolution and cannot produce tomographic images due to photon attenuation within tissues [10]. However, only one recent study has used this technique for cell graft monitoring in the brain [11]. Hence the sensitivity of this kind of application still requires further definition.

The study of post-graft cell survival and fate over a long period is crucial. Nevertheless, gene therapy experience indicates that foreign gene expression by eukaryotic cells can be rapidly shut down. Several reports have shown that viral promoters like CMV, commonly used to drive gene expression, are rapidly inactivated [12,13]. This is known to be partly due to the methylation of CpG islands present in the viral promoter sequence [14–16]. These mechanisms strongly reduce the overall efficiency and longevity of transgene expression and would therefore certainly limit the long-term monitoring of cell grafts in the case of molecular imaging. Several studies have shown that optimisation of the DNA sequence as a CpG-free sequence and the use of mammalian promoter [17–19] can increase long-term transgene expression and should therefore be considered in molecular imaging strategies.

As discussed below, scalp attenuation is an important limitation in *in-vivo* brain imaging. The aim of this pilot study was to evaluate the feasibility and sensitivity of [^18^F]FHBG/HSV1-TK molecular imaging in monitoring cerebral grafted cells. In order to promote long term HSV-1TK expression, we developed a stably transfected neuronal cell line for this work with a CpG-free sequence containing HSV1-thymidine kinase cDNA with an optimised mammalian codon sequence controlled by the human EF1alpha promoter. This non-silenced promoter has yielded good results in terms of gene expression in a wide range of mammalian cells [20,21] including neuronal cells [22]. Using a cell calibration range of these HSV1-TK-expressing cells pre-incubated with the radiotracer [^18^F]FHBG and PET imaging both in cell culture and *in vivo* after brain injection, a perfectly linear correlation between the PET signal and number of grafted cells is highlighted with no signal attenuation using CT correction. The [^18^F]FHBG/ HSV1-TK molecular imaging system could then be used to monitor intracerebrally grafted cells.

## Materials and methods

### FHBG radiosynthesis

[^18^F]FHBG synthesis was adapted from the method described previously [3]. Briefly, [^18^F]FHBG was produced on a Raytest® synthesis module by nucleophilic substitution using tosyl-FHBG (ABX). as a precursor. [^18^F]Fluoride is produced by the cyclotron (Cyclone 10/5 IBA) via the ^18^O (p, n) ^18^F reaction. After azeotropic drying, the precursor was heated for 20 min at 110°C. The reaction mixture was then cooled and added to the hydrolysis solution (HCl 1M, Merck) and heated for 5 min at 115°C. The reaction mixture was then neutralised by adding 2 M NaOH and 0.5 M trisodium citrate solutions (Cooper). HPLC purification was carried out in a semi-prepared Bischoff column (Prontosil®, L 250 mm, ø 10 mm, pores 5 μm) with a mobile phase consisting of absolute ethanol (WRS pharma prolabo) /sodium acetate (0.1M, Cooper) mixture (10/90 v/v). The [^18^F]FHBG retention time was 17.52 min, with a flow rate of 2 ml/m. Specific activity was superior to 3.2 GBq/μmol. 100% of radiochemical purity was not observed until 4 h after radiosynthesis. Radioactivity concentration was always superior to 350 MBq/ml with a low quantity of ethanol (< 10%) for experimental use on biological materials (*in-vitro* cell incubation or animal experiments). After medium dilution, [^18^F]FHBG incubation medium for cellular uptake always contains less than 0.001% of ethanol.

### Generation of stable Neuro2A-TK cell line

The mammalian expression vector pCpGfree-HSV1-TK was obtained from Invivogen (Toulouse, France). It is a CpG-free plasmid containing HSV1-thymidine kinase cDNA with an optimised mammalian codon sequence. cDNA is controlled by the human EF1alpha promoter and a murine CMV enhancer. The plasmid was amplified with the appropriate bacterial strain (*E*. *coli* GT115) for CpG- free plasmid and prepared using an endotoxin-free plasmid kit (Macherey-Nagel NucleoBond^®^ Xtra Maxi EF) to avoid the presence of possible inflammatory contaminants.

**Fig 1:**
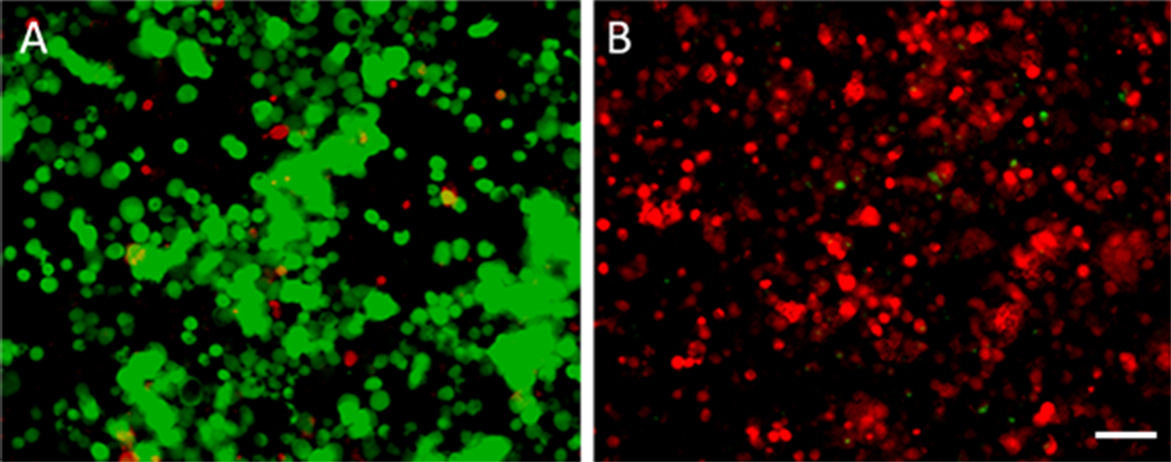
Generation of stably transfected Neuro2A -TK cell line. Neuro2A cells were transfected with the optimised mammalian vector pCpG-free-HSV1-TK. Stably transfected cells were selected by antibiotic pressure (G418, 600μgr/ml) over a 3-week period. The selection procedure was confirmed with the fluorescent live–dead staining assay on stably transfected Neuro2A-TK cells (A) or Neuro2A control cells (B). Red staining indicates dead cells; green staining indicates live cells. Scale bar 30 μm.

Neuro2A cell line (ATCC) was cultured in Dulbecco’s modified Eagle medium (DMEM) supplemented with 10% foetal bovine serum (FBS), and 1% penicillin-streptomycin at 37°C in a humidified atmosphere containing 5% CO_2_. For transfection, 7 X 10^5^ cells were seeded into 6-well tissue culture dishes and allowed to adhere overnight. The next day, DNA-lipid complexes were formed with 2.5 μg pCpG-free-HSV1-TK plasmid using Lipofectamine™ LTX and PLUS™ Reagents, following the manufacturer's instructions (Invitrogen, CA USA) and added drop-wise to the cells. The cells were incubated at 37°C and 24 h later, growth medium supplemented with G418 (600μgr/ml) was added for selective pressure. After 3 weeks of selection, drug-resistant colonies were removed from the plates and expanded to generate polyclonal populations of Neuro2A cells stably expressing HSV-1 TK. The fluorescent live–dead staining assay (Molecular probe) was used to confirm the selection procedure (Fig 1). Cytotoxic assay using Ganciclovir (Invivogen, Toulouse) was performed to also validate TK expression in the stably transfected Neuro2A-TK cell population.

### [^18^F]FHBG incubation procedure with neuro2A-TK cells

#### *In-vitro* optimisation of [^18^F]-FHBG cell uptake

To test different incubation periods and activities of [^18^F]FHBG, 1X10^6^ Neuro2A-TK cells or control Neuro2A cells were seeded in 6-well tissue culture dishes with 3 ml of growth medium the day before the experiment, and allowed to adhere overnight. The next day, 115 kBq/ml or 555 kBq/ml of [^18^F]FHBG were added to each test well. Variations between incubated activities were accepted due to the low activity used and radioactivity manipulation (adhesion to side of syringe, etc.). Cells were incubated at 37°C to promote [^18^F]FHBG uptake for 1, 2 or 3 hours, as indicated. After the incubation period, cells were washed 3 times for 10 minutes à 37°C with 3 mL of medium. Cells were then removed from their support using 1 mL of trypsin//EDTA (Gibco-Brl), and neutralised with 2 mL of medium. 3 ml of cell suspension were collected and counted with a gamma counter (PERKIN ELMER 1480 gamma WIZARD counter). The total cell activities, corrected from the counting efficiency apparatus (48%), were then expressed as disintegration per minute (dpm).

For each experiment, to determine the percentage of [^18^F]FHBG cell retention, the exact total deposited activity of [^18^F]FHBG were calculated by counting 3 ml of a 1/100 dilution after the same incubation time (Total deposited activity). Background radiation (BR) was determined with 3 mL of culture medium. The percentage of [^18^F]FHBG cell retention was then calculated for the different incubation procedure, using the following equation:

[18F]FHBGcellretention=((dpmcells−BR)/(Totaldepositedactivity))×100

No loss of viability was observed under these conditions, as confirmed by the trypan blue exclusion assay (98±1% of viability, n = 3) All of these *in-vitro* experiments were performed in at least three independent experiments for the two groups of cells.

#### Cell calibration range counter or PET/CT study

For the cell calibration range study, 5 X 10^6^ of Neuro2A-TK cells or control Neuro2A cells were seeded in 100- mm cell culture dishes in 10 mL medium and incubated the next day with 555Bq/ml of [^18^F]FHBG. After incubating for 3 hours at 37°C, cells were washed 3 times with 10 mL of medium and cell suspensions were prepared as described above. After centrifuging (10 min at 800 rpm) to concentrate the cells, cell suspensions were counted with a haemocytometer (1 X 10^4^ to 3 X 10^6^ cells). Cells were either diluted in 3 mL medium and counted with a gamma counter (as described previously) or deposited in a 96-well plate in a total of 200 μl medium for PET/CT imaging. In order to compare experiments, results were reported as decay-corrected disintegration per minute (dpm) for a delay of 5 h after incubation.

To really compare cell activities, the same cell preparations were used for gamma counter, PET/CT or for implantation experiments of [^18^F]FHBG Neuro2A-TK cells. We carried out these calibration ranges using the two measurement techniques (PET and counter) during 5 independent experiments (n = 5).

### Animals,M1 cortical lesion induction and [^18^F]FHBG neuro2A-TK cell transplantation

All of the animals (*N* = 22), were housed and treated according to guidelines issued by the Council of the European Communities. This protocol was approved by the « Direction départementale de la Protection des Populations de la Haute-Garonne (authorisation no. 31125507) » and the « Comité d’éthique pour l’expérimentation animale Midi-Pyrénées ». All efforts were made to minimize the number of animals used, and to avoid suffering. Adult Sprague-Dawley rats (300-350 g, Elevage Janvier, Le Genest-St-Isle, France) were anaesthetised with ketamine/medetomidine (36/0.47 mg/kg, intraperitoneal injection) and premedicated with methylprednisolone 0.4 mg/kg.

Cortical lesions focused on the caudal forelimb motor area (M1) were induced by malonate stereotaxic injection (3M solution, pH 7.4 in PBS; Sigma-Aldrich, France) [23] into the left hemisphere at the following stereotaxic coordinates: 2.5 mm lateral to Bregma, and 2 mm deep (Paxinos and Watson, 1998). Malonate, a toxin inhibitor of a mitochondrial enzyme, was injected at a rate of 1 μL/min. After infusion of 5 μl, the cannula was left in place for 5 min to allow complete diffusion of the injected solution. The incision was closed and the rats were allowed to recover in their normal environment. Rats were housed in pairs on a 12h:12h light:dark cycle and fed standard rat chow and water ad libitum. During housing, animals were monitored twice a day for health status during 3 days post-surgery and then daily. Two weeks later, the animals displayed neurological dysfunction [23]. A second surgical intervention was then performed to implant various quantities of [^18^F]FHBG labelled Neuro2a-TK cells. The cells were pre-incubated with [^18^F]FHBG and prepared as described above just prior to transplant to ensure optimal cell viability and signal. The anaesthetised rats were then placed in a stereotaxic frame and the lesioned area of each animal (Bregma: A: 0 mm, L+2.5 mm, V+2.0 mm) was injected slowly (1μl/min) with 5 μl of [^18^F]FHBG labelled Neuro2a-TK cell suspension using a Hamilton micro syringe fitted with a 22G needle. 19 rats were transplanted with different quantities of [^18^F]-FHBG labelled Neuro2a-TK cells in five independent experiments: 1 X 10^4^ (n = 1); 5 X 10^4^ (n = 2); 1 X 10^5^ (n = 3); 2,5 X 10^5^ (n = 1); 5 X 10^5^ (n = 3); 1 X 10^6^ (n = 4). A withdrawal criterion was applied: rats with signal localisation outside the brain were excluded from the quantification study (n = 5).

### Cell and small animal PET imaging

The *in-vitro* cell calibration range and rats grafted with [^18^F]-FHBG labelled Neuro2a-TK cells were acquired under the same conditions. Acquisitions were performed under anaesthesia (ketamine/medetomidine, 36/0.47 mg/kg, intraperitoneal injection) 15 to 30 min and 2h after the labelled grafted cells. Acquisition was performed in list mode with PET/CT imaging (Siemens Truepoint biograph) and lasted 15 min (5-mm FWHM Gaussian filter, 3 repeats, 21 subsets). All images were automatically corrected for radioactive decay due to manufacturer software settings. Following the reconstruction, the CT images were spatially aligned to match the PET images. In addition to image reconstruction, the CT data were used for attenuation correction of PET images. Animals were euthanised just after PET imaging by intraperitoneal injection of pentobarbital (200mg/kg).

### Image analysis and quantification study

The processing of reconstructed images was performed using Sisyphe software [24]. Volumes of interest (VOI) were drawn in PET images from grafted rats or cells on a 96-well plate. Large VOI were used to record all signals and actually estimate total activities from cells. To quantify each VOI signal, the following calculation was performed: mean activity of VOI * VOI volume * rescale slope factor. This activity (Bq/ml) was converted into dpm and results were expressed as dpm activity for the number of [^18^F]FHBG Neuro2A-TK cells used in the experiment. To compare all experiments, decay corrections were used to obtain activity 5 h after starting [^18^F]FHBG incubation on Neuro2A-TK cells.

#### *In-vivo* labelling by intracerebral injection [18F]FHBG after cell graft

Neuro2A-TK cells (1 or 3 X 10^6^) without [^18^F]FHBG pre-incubation were injected in animals as described above (n = 2). One day later, 1110 kBq of [^18^F]FHBG in a maximum of 5 μl were injected intracerebrally at the rate of 1 μl/min with the same surgical procedure as that used for cell grafts. This activity corresponds to the incubation of 555 kBq/ml *in vivo* as the volume of rat brain is considered to be approximately 2 mL [25]. A control rat without any cell graft was also prepared (n = 1). Acquisitions were performed 90 and 160 min after [^18^F]FHBG injection with the same parameters as described above. The number of dpm was calculated for each conditions after decay correction and subtraction of the activity observed in the control rat brain. The percentage of injected dose per gram of tissue (% ID/g) in the grafted area was then calculated.

#### Statistical analysis and sensitivity threshold

Data were expressed as the mean +/- standard deviation. Statistical analysis between two independent groups was performed using two-tailed Student’s t test with Xcel stat software. Results were considered significant for p values below 0.05.

All signal values below 2 standard deviations of the noise were not considered as specific signals.

## Results

To investigate the option of monitoring and evaluating cell graft survival using the [18F]FHBG/ HSV1-TK molecular imaging system after cerebral injection, a neuronal cell line stably expressing HSV1-TK (Fig 1) was developed using a DNA sequence especially designed to ensure long-term expression of HSV1- thymidine kinase in mammalian cells and avoid methylation-driven silencing of transgene expression.

### [18F]FHBG incubation parameters

#### Time period and optimal activity

**Fig 2:**
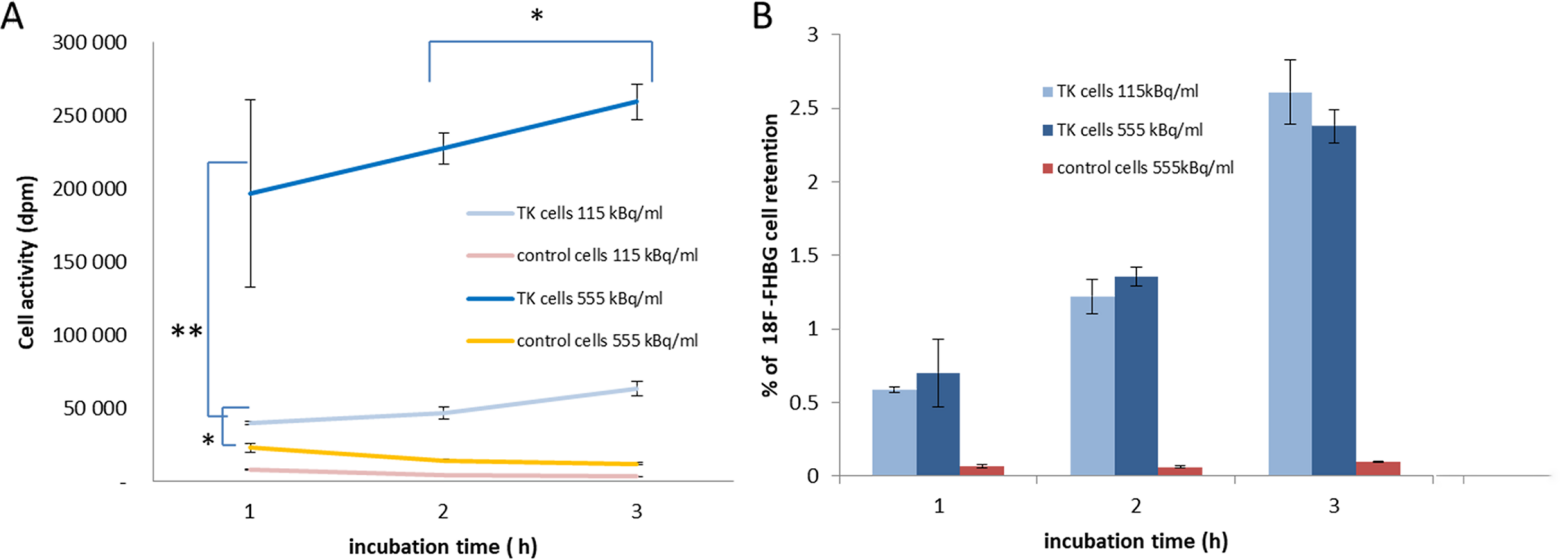
Cell radioactivity retention. (A) Cell activity (dpm) or (B) Percentage [^18^F]FHBG cell retention as a function of the incubation time with 115 or 555 kBq/ml of [^18^F]FHBG. Each value represents the mean ± SD (* p<0.05, ** p<0.005). A significant difference was observed between the activity retained by the control cells and that retained by Neuro2A-TK cells in terms of [^18^F]FHBG concentrations and incubation time.

To ensure the optimum signal from radiolabelled cells, incubation parameters were studied to promote [^18^F]FHBG uptake. A fixed number of Neuro2A-TK or control Neuro2A cells were incubated with different activities of [^18^F]FHBG at different times (Fig 2). Counter analysis showed a difference between [^18^F]FHBG uptake by TK-expressing cells and control cells just 1 hour after incubation for the two activities tested with this difference increasing after 2 h and 3 h (p< 0.001, Fig 2A). Maximum cell activity was observed for incubation of 555kBq/ml over 3 h. This value was significantly higher than that recorded with 115 kBq/ml after 3 hours’ incubation (p <0.0001) or for 2 h with 555 kBq/ml (p = 0.02). Cell retention of [^18^F]FHBG at 3 h with 555 kBq/mL with Neuro2A-TK cells was 25 fold higher than that observed with control cells (2.37+/- 0.11% for TK-expressing cells and 0.09 +/- 0.01% for control cells, Fig 2B). Incubation with an activity concentration of approximately 555 kBq/ml over 3 h seems the most efficient parameter for cell uptake and was used for the rest of the study.

#### Counter and PET/CT cell calibration range

**Fig 3:**
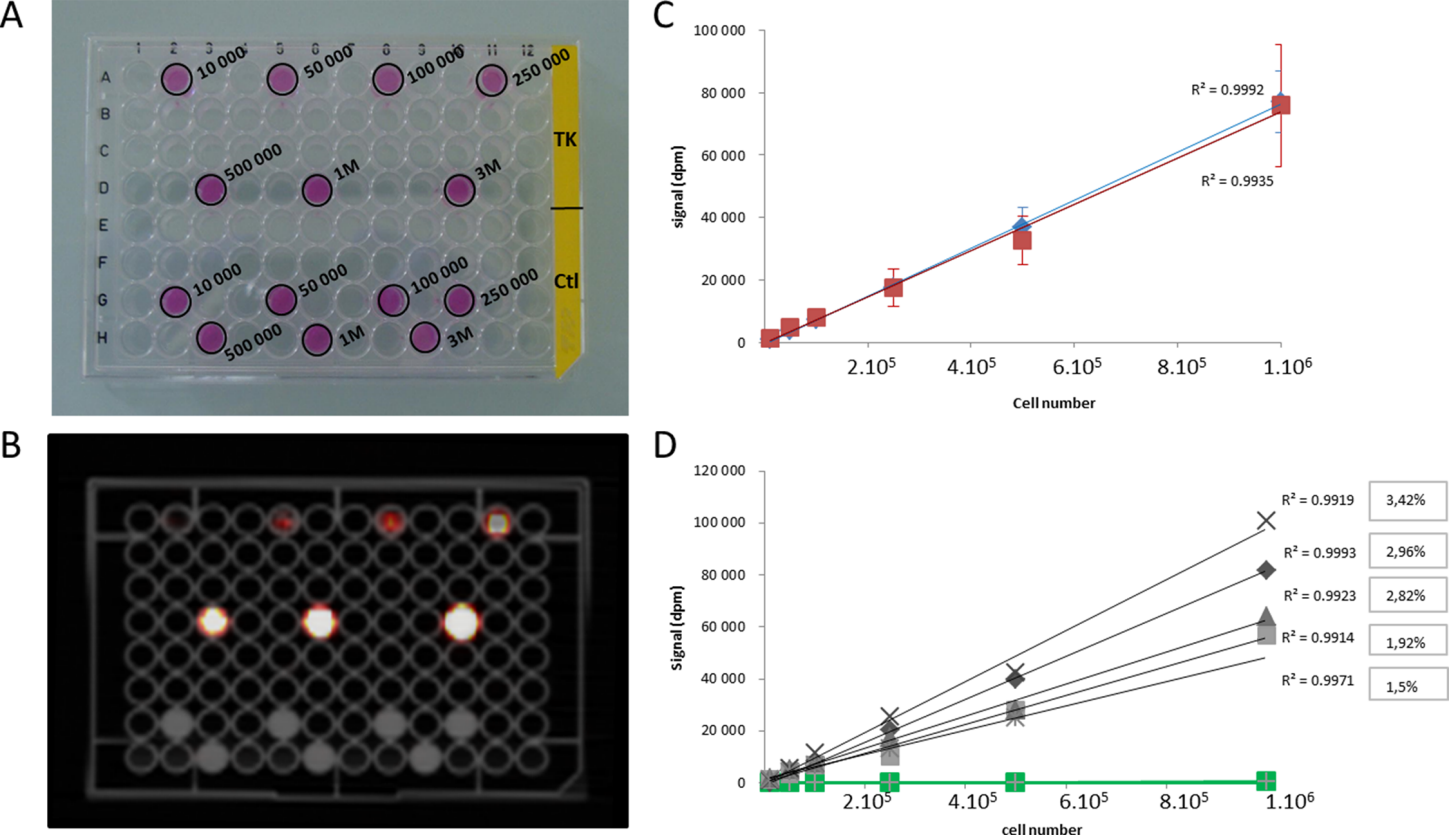
Cell calibration range using the gamma counter or PET/CT camera. A 96-well plate containing different quantities of [^18^F]FHBG labelled Neuro2a-TK cells and control cells were imaged using a PET/CT camera (A, B). The decay-corrected signals expressed in dpm were calculated (C) for the different cell quantities using the gamma counter (blue) or the PET/CT images (red) (n = 5 experiments on separate days, p = 0.626 no significant difference was observed between average counter titration and average camera titration). Different camera calibration ranges for [^18^F]FHBG labelled Neuro2a-TK cells in grey (n = 5) and for control cells in green were performed (D). The percentage of [18F]FHBG cell retention was also calculated for each independent experiment (grey square).

The activities of various quantities of [^18^F]FHBG labelled cells were evaluated using optimum incubation parameters for cell uptake using a gamma counter or PET/CT camera (Fig 3A and 3B). As shown in Fig 3C (blue curve, gamma counter calibration range), there is a linear proportionality between [^18^F]FHBG uptake and cell number (r^2^ = 0.999). The number of cells expressing TK can therefore be evaluated based on the signal measured in dpm on the gamma counter. The same cell quantity range was analysed by PET/CT. Signals were not homogenous on each well. This was perhaps due to cell precipitation and clumping during the acquisition period and to the partial volume effect. Large VOI were calculated for each well taking all signal data into account. The average of VOI was 6 458 +/- 1 690 mm3. Signal proportionality with cell number was also very good after analysing PET/CT images (r^2^ = 0.994; n = 5, Fig 3D). The variability in experimentation observed between the calibration ranges seems in fact correlated more with cell uptake variability in routine experiment than with image signal analysis. A comparison between camera counting and counter counting shows that there is no significant difference between the two measures.

For PET/CT experiments, the average non-specific signal was 921 dpm +/- 807 corresponding to 15 526 +/-10 910 cells (according to the cell calibration range). Hence, due to noise variability according to the cell calibration range, the sensitivity threshold of this technique was around 2,5 X 10^4^–5 X 10^4^ cells (equivalent to two standard noise deviations). These results were confirmed by t test analyses between signals of grafted cells (n = 5 per quantity of grafted cells) with camera and background (n = 5) which show a significant difference (p <0.005) for all quantities of cells tested except for 1 X 10^4^ cells (p = 0.03). This test confirms that sensitivity is between 1 X 10^4^ and 5 X 10^4^ cells.

### *In-vivo* cell monitoring and quantification after [^18^F]FHBG Neuro2A-TK cell transplantation

**Fig 4:**
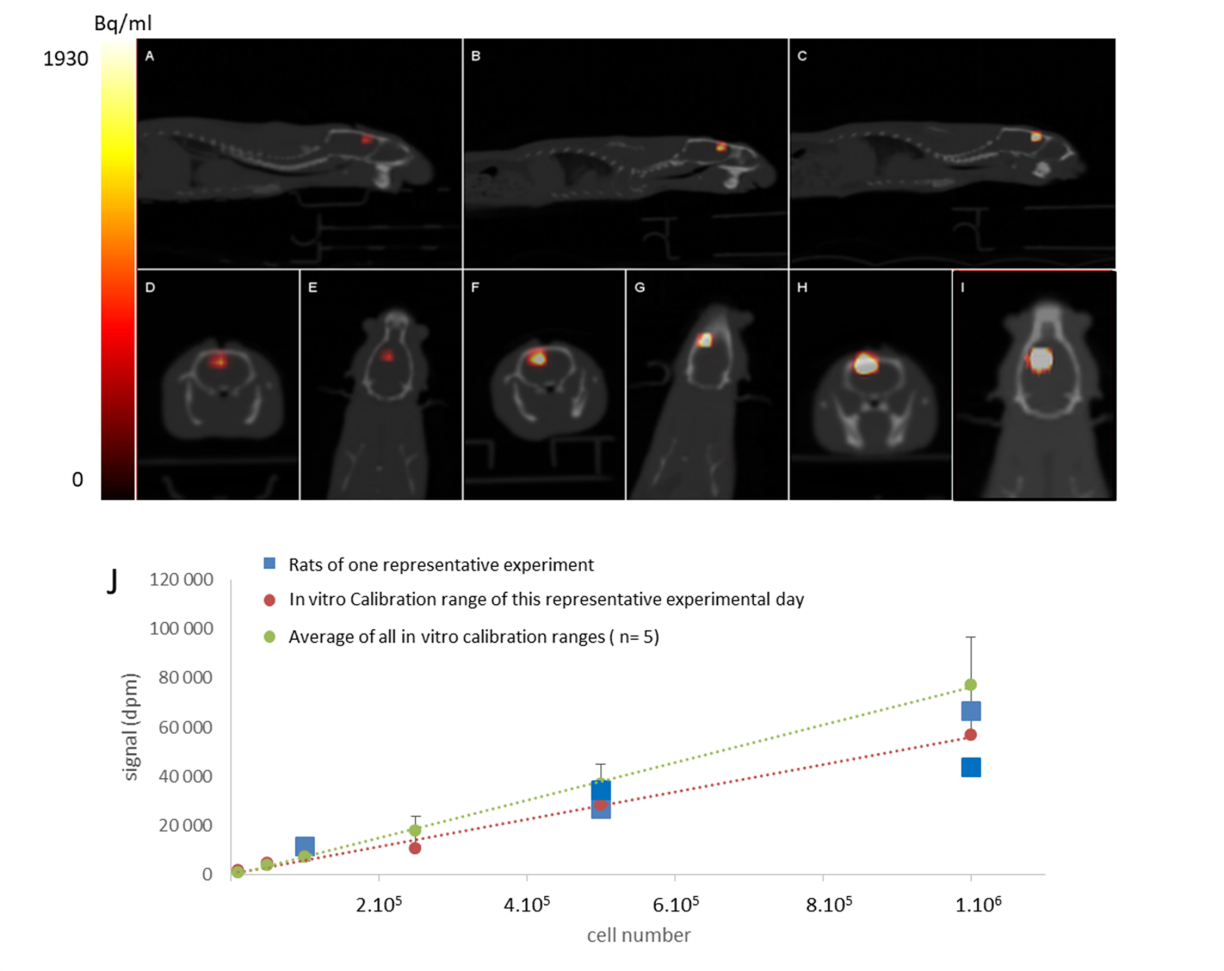
*In-vivo* cell monitoring and signal quantification in rat brain. Representative PET/CT images in sagittal [A,B,C], coronal [D,F,H] and axial [E,G,I] axes of rat grafted with different quantities of [^18^F]FHBG labelled Neuro2a-TK: 1 X 10^5^ cells [A,D,E] 5 X 10^5^ cells [B,F,G] and 1 X 10^6^ cells [C,H,I]. Representative experiment with decay-corrected signal of [^18^F]FHBG labelled Neuro2a-TK cell-grafted rats (5 rats imaged on the same day, blue square), using the daily cell calibration range (red dots) and the average cell calibration ranges (n = 5 experiments, green dots with standard deviation) imaged with the camera (J). Colour scale intensity (Bq/ml).

**Fig 5:**
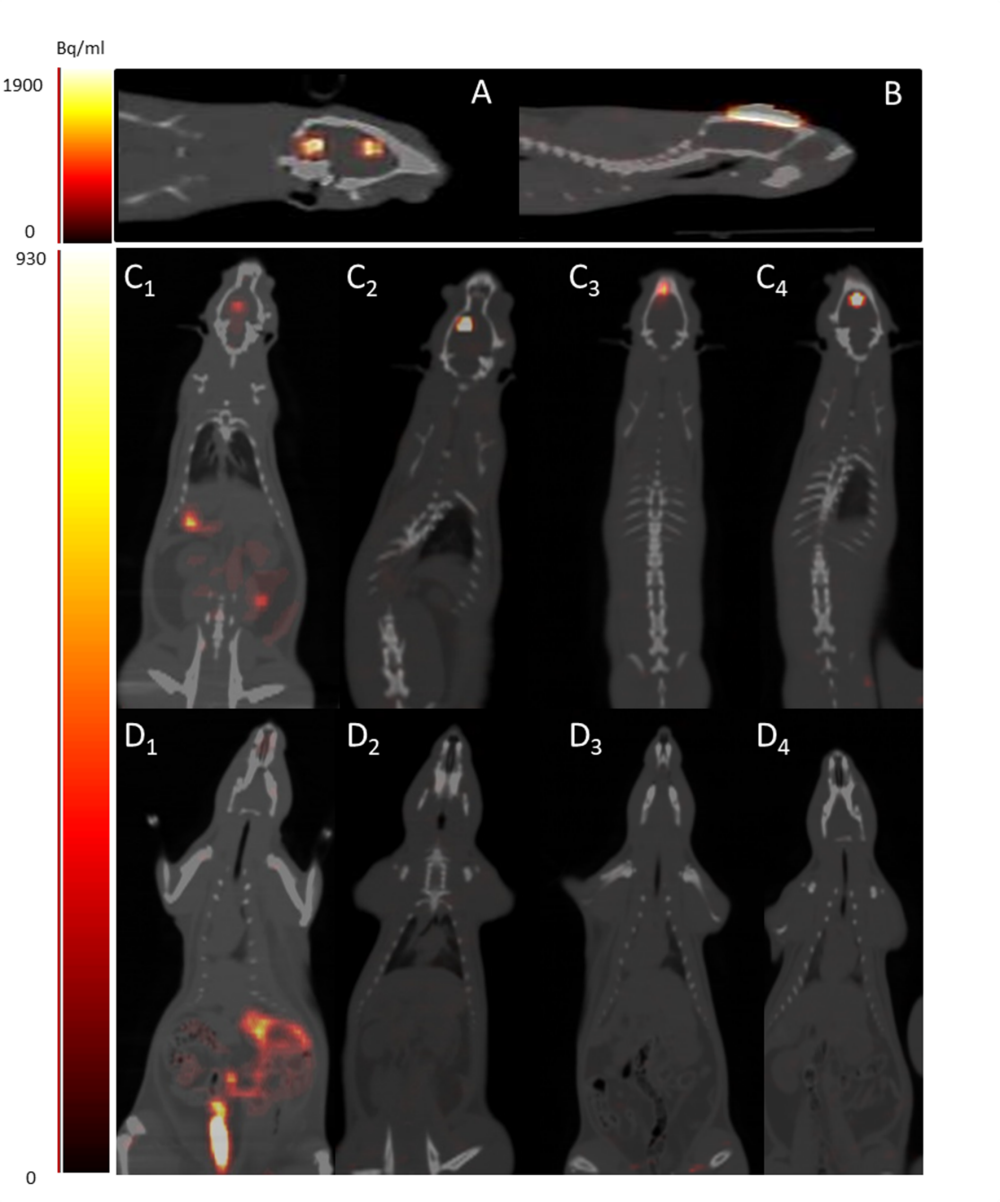
Quality control of graft process by PET imaging. PET/CT used to monitor quality and localisation of grafted cells. During the graft process, cells could reflux on CSF cisterna due to an excessive injection volume (n = 2, example on A) or could be ejected from the injured area and released onto the scalp due to the incorrect positioning of the needle (n = 3, example on B). Comparison of [18F]FHBG rat biodistribution 2h after intracerebral injection in the lesioned area of the radiotracer alone (rat 1 image C_1_ and D_1_, 2 axial sections of the same rat) or of [18F]FHBG labelled Neuro2A-TK cell (rat 2,3,4, 2 axial sections respectively on C_2,3,4_ and D_2,3,4_). Color scale intensity (Bq/ml).

To validate this methodology *in vivo*, Neuro2A –TK cells were pre-incubated with [^18^F]FHBG and directly injected intracerebrally into rats with cortical lesions. PET/CT images of the rats were taken immediately (Figs 4 and 5). Fig 4A–4I shows representative PET/CT images of rats grafted with different quantities of cells. When the injection is administered under good conditions, namely a small volume of less than 5 μl and a slow rate of injection, a signal is only localised at the cell injection site. However, the injection of an excessive volume into the lesion could cause cell reflux on CSF cisterna (Fig 5A). Fusion with CT also ensures control if the graft is localised in an injured area. As shown in Fig 5B, cells could be ejected from the injured area and could diffuse on the scalp due to various causes (internal excessive pressure, misplacement of the needle, head movement after injection cells). This underlines the importance of monitoring the injection to ensure graft quality. The withdrawal criterion was so applied and 5 rats were excluded from the quantification study for signal localisation outside the brain.

Quantitative analyses of brain rat signals and comparisons with the PET/CT calibration range (Fig 4) highlight a perfect correlation between brain signal and the number of injected cells (linear correlation r^2^ = 0.98 with n = 14 grafted rats in five independent experiments). To test the most reliable methodology for quantifying cells from the signal obtained from rat PET/CT images, *in-vivo* signals were compared to an average in vitro cell calibration range (n = 5 independent experiments) or with the calibration range of the day (same cell solution used for the graft) (Fig 4J). The two calibration ranges were distinct. There was less variability with the calibration range of the day than with the average calibration range per se, p = 0.71 and p = 0.56 respectively, n = 14 animals).

The distribution of [^18^F]FHBG alone after intracerebral injection in injured rats was studied for 2 h (Fig 5C and Fig 5D). After intracerebral injection in the lesioned area, [^18^F]FHBG uptake was noted in several organs: spleen, intestinal tract, kidney and bladder (Fig 5C_1_ and Fig 5D_1_). This corresponds to normal radiotracer distribution [26]. This distribution was then compared to the tracer kinetic obtained with [^18^F]FHBG labelled Neuro2A-TK cell-grafted rats (Fig 5C_2,3,4_ and Fig 5D_2,3,4_). No uptake of [^18^F]FHBG was observed in any organs even 2 h after injection of [^18^F]FHBG labelled Neuro2A-TK cell-grafted rats (n = 3). The signal was only localised in the grafted area and confirmed that no [^18^F]FHBG or [^18^F]metabolite was released during graft procedure and 2 h hours later due to cell lysis or other phenomenon.

### *In-vivo* labelling for cell graft monitoring

**Fig 6:**
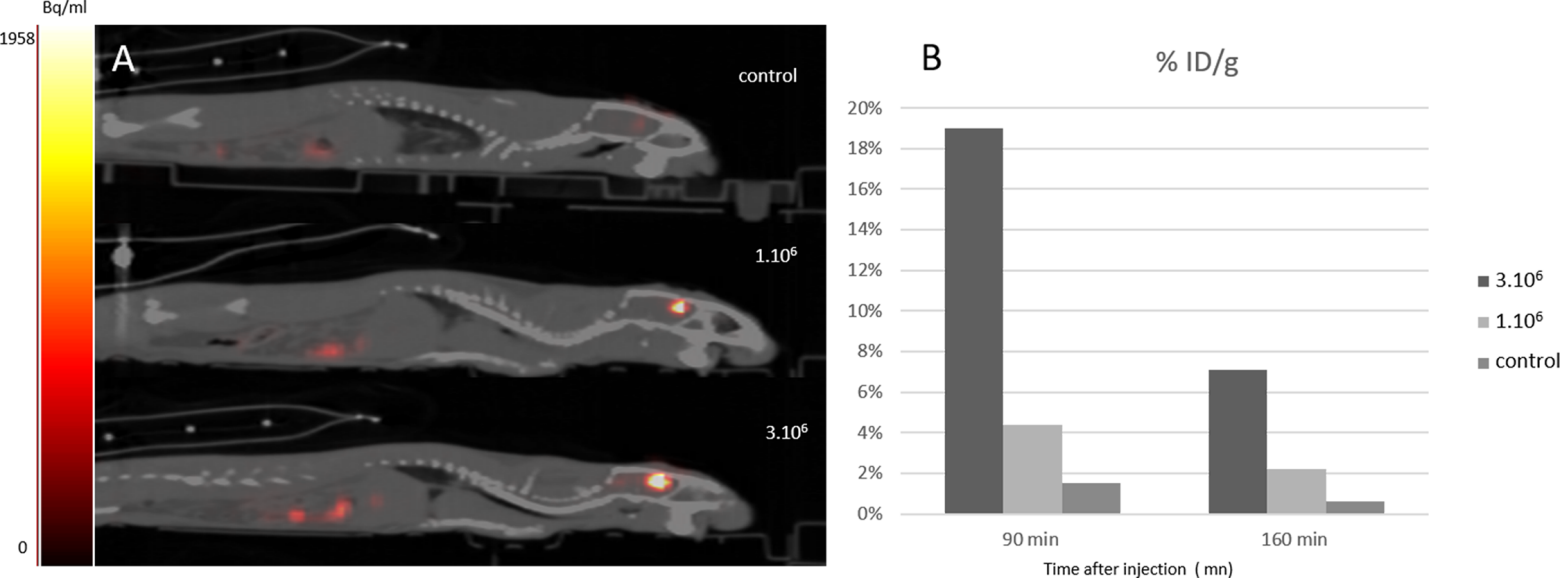
*In-vivo* labelling by intracerebral injection [^18^F]-FHBG after cell graft. 1110 kBq of [^18^F]FHBG were injected intracerebrally one day after the graft (1 X 10^6^ or 3 X 10^6^ cells, n = 1 per quantity of cells)) or in the control rat (injured but not grafted n = 1). 90 and 160 min after injection, PET /CT images showed a whole body distribution of the radiotracer and cerebral retention into the grafted area (A). Image analyses were performed and the percentage dose injected per gr of tissue (% ID/g) was calculated for the different time and cell quantity(B). 90 and 160 min after injection, a higher % of ID/g was recorded in rats with 3 X 10^6^ grafted cells than those with 1X 10^6^ grafted cells.

To evaluate the option of labelling TK-expressing cells *in vivo* and performing *in-vivo* cell graft monitoring, Neuro 2A-TK cells were grafted and [^18^F]FHBG was then injected. The tracer was injected intracerebrally to ensure optimum cell accessibility. PET data 90 and 160 minutes after injection showed a whole body distribution of the radiotracer with a hyper signal in the rat brain injected with Neuro2A-TK cells compared to the control rats (Fig 6A). The control rat brain signal was stable between 90 and 160 min and equivalent to around 0.5–1.5% of the [^18^F]FHBG dose injected. Retention of [^18^F]FHBG was observed at the cell grafted site up to 160 min after injection (Fig 6B). 90 min and 160 min after injection, grafted area retention is clearly higher in rat with 3 X 10^6^ grafted cells (respectively 18.9% and 7.08% of ID/g) than with rat containing 1 X 10^6^ of grafted cells (respectively 4.37% and 2.2% ID/g). This study was a pilot study but the % dose injected shows proportionality between the number of cells and the signal retained in the grafted area and could then allow semi-quantitative estimation of the number of cells.

## Discussion

Regarding advances in transplant biology and regenerative stem cell therapy, there is an increasing need to monitor and understand the behaviour of various exogenously added cells in patients. Reporter genes such as HSV-TK should perhaps be considered as adjuncts to any clinical cell therapy trial in order to exploit this safety potential (by adding ganciclovir for the suicide gene strategy) and to longitudinally monitor cell graft function and fate. In this paper, we show the feasibility of using the HSV1-TK/[^18^F]FHBG molecular imaging system to monitor and above all evaluate cell graft semi-quantitatively after intracerebral injection. To our knowledge, only one other study has used this kind of molecular imaging for brain graft tracking after brain injury [11].Many factors are involved in selecting the optimal reporter gene expression system which could impact the sensitivity of the technics. For long-term graft monitoring, the transgene should be expressed for as long as possible with good expression for an optimal sensitivity threshold. Virus-derived promoters such as CMV generate an important gene expression and could therefore provide a good signal when coupled with a probe. However, as these promoters have the disadvantage of being rapidly inactivated, the mammalian promoter with the CpG-free sequence vector was chosen, sometimes leading to lower but prolonged expression [13,20]

For *in-vitro* experiments, comparison of the two concentrations of [^18^F]FHBG incubation solution showed an increase in signal between the two doses with, however a comparable [^18^F]FHBG cell retention percentage showing that HSV-TK enzymatic activity is not saturated in this concentration range. However, no problem of cell viability due to the high dose of radioactivity was observed. The highest concentration was chosen to increase the sensitivity of the technique and facilitate the location and magnitude imaging of probe accumulation. Other teams typically use 150 kBq/ml for incubation [27]. In fact, with these conditions (higher activity concentration, mammalian promoter, CpG-free sequence, optimised mammalian codon sequence), we evaluated and showed that *in-vivo* sensitivity was around 5 X 10^4^ cells detected with PET/CT. With different promoters and activity concentrations used, other teams have highlighted an i*n-vivo* sensitivity of 1 X 10^5^ cells at best using subcutaneously implanted cancer cells [28,29,30]. The next step will be to study TK expression stability using this plasmid construction in long-term studies to ensure in-vivo cell monitoring over a prolonged period.

A successful imaging system requires sufficient signal sensitivity to detect grafted cells and sufficient spatial resolution to determine their location within the tissue/organ, especially in the brain which could induce a strong decrease in signal due to scalp attenuation. This pilot study has shown that cell injection quality could be controlled with PET/CT image of [^18^F]FHBG labelled Neuro2A-TK cell-grafted rats. The data presented in this article demonstrate that PET/CT imaging possesses sufficient spatial resolution to select animals receiving cells in good conditions, to evaluate viability of grafted cells and provide initial graft data. With CT correction attenuation, PET data were corrected from scalp attenuation and then matched perfectly with data obtained with the *in-vitro* cell calibration range showing that *in-vivo* semi-quantification is possible using a calibration range of the same cell type. Uptake stability was also established and no radioactivity efflux occurred as observed with the hNIs/Tc reporter probe system [31,32].

The final aim of the molecular imaging system will be to facilitate longitudinal monitoring of cell grafts in contrast to histological techniques. With the HSV-TK reporter system for imaging living cells in the brain parenchyma, the relatively low permeability of [^18^F]FHBG through the BBB (blood brain barrier) [26,33–35] can be a limiting factor. After stroke, there is potentially a disruption in the barrier function but the amplitude and opening duration of BBB seems quite variable and certainly depends on the location and size of the lesion [36,37], which complicates longitudinal quantitative study using [^18^F]FHBG. One other team recently referred to cerebral stem cell graft monitoring [11], and confirmed that [^18^F]FHBG crosses the BBB only if it is disrupted.

In this report, we have tested radiotracer injection directly into the brain, not intravenously, in order to develop the technique and overcome any potential issue with [18F]FHBG penetration through the BBB. However, as the intracerebral approach is rather invasive for longitudinal studies, future studies would focus on testing other routes of delivery. Intravenous administration with, for example, a brain-focused ultrasound technique to temporarily open the BBB could be an alternative [38–41]. Development of PET tracer with improved brain penetration across the blood brain barrier could also figure in future strategies [42]. *In vivo*, it would be difficult to guarantee the volumic activity of incubation in the brain because distribution and cerebral elimination of the tracer takes place. However, from the incubation parameter study presented in this article (Fig 2), the penetration of 1 110 kBq into rat brain (theoretically equivalent to 2ml volume) would be necessary to obtain the optimised radiotracer concentration of 555 bq/ml. If we consider a radiotracer penetration of only 3% after intravenous injection, a dose of 37 MBq would be necessary, which is an acceptable activity to inject.

It is obvious that developing safer, more effective and accurate imaging methods is crucial to enhance our understanding of cell-based stem therapy. Firstly in the case of animal studies, however the potential to gather safety and general biological information by directly imaging the grafted cells in patients taking part in cell therapy clinical trials should be added to these considerations. Because preclinical models do not always reflect the biology of either healthy humans or patients, it is important for new tools to be applicable for both humans and animal models. Different radioactive compounds are routinely injected in patients for clinical diagnosis. [^18^F]FHBG appears to be an effective tool for obtaining biological information in a non-invasive manner and has been already tested in a clinical trial, in patient with leukaemia after infusion of Tcell genetically modified [7,8].

## Conclusion

In this report, we show the feasibility to monitor TK-expressing cells with [^18^F]FHBG reporter probe after brain graft. Long-term studies are still required. The ability to track cells *in vivo* after transplantation in the same animal over time will improve our understanding of the fate of grafted cells, thereby minimising the number of animals needed to determine the optimal cell source, the best injection method and ideal timing to produce genuine functional recovery post-graft. Inevitably, this type of method would significantly shorten the time between pre-clinical and clinical investigations with the emphasis on rapid optimisation.

## Supporting information

S1 File Arrive guidelines checklist. (PDF)
